# Productive and Physiological Response of Male Rabbits to Dietary Supplementation with Thyme Essential Oil

**DOI:** 10.3390/ani10101844

**Published:** 2020-10-10

**Authors:** Ahmed A. A. Abdel-Wareth, Abdallah E. Metwally

**Affiliations:** 1 Department of Animal and Poultry Production, Faculty of Agriculture, South Valley University, Qena 83523, Egypt; a_bkr1@yahoo.com; 2 Department of Nutrition and Clinical Nutrition, Faculty of Veterinary Medicine, Zagazig University, Zagazig 44511, Egypt

**Keywords:** blood biochemistry, performances, rabbits, semen quality, thyme essential oil

## Abstract

**Simple Summary:**

The present study was carried out to compare the potential effects of the levels of thyme essential oil on the productive performance and serum metabolic profile of male rabbits. Rabbits were assigned to five dietary treatments including a basal diet as a negative control, a basal diet supplemented with an antibiotic as a positive control, and a basal diet supplemented with 60, 120, or 180 mg/kg of thyme essential oil. The main results showed that the levels of thyme essential oil contributed to the improvement of productive and the physiological response compared with the negative and positive control of male rabbits.

**Abstract:**

The present study aimed at assessing the efficiency of thyme essential oil (TEO) as an alternative to antibiotics for improving the productive performance and serum metabolic profile of male rabbits. A total of one hundred and fifty 70-day-old male Californian rabbits were assigned to five dietary treatments, including a basal diet as a negative control (NC), a basal diet supplemented with an antibiotic as a positive control (PC), and a basal diet supplemented with 60, 120, or 180 mg/kg of TEO. The experiment period lasted for 60 days. Supplementation of TEO levels significantly (*P* < 0.01) increased daily body weight gain and improved feed conversion ratio of male Californian rabbits compared to NC and PC groups. Similarly, the TEO remarkably enhanced the semen characteristics of rabbits compared to NC and PC groups. Supplementation of TEO significantly decreased aspartate transaminase, alanine transaminase, urea, and creatinine compared with NC and PC groups. Supplementation with TEO increased serum testosterone concentration compared to NC and PC treatments. Our data demonstrate that TEO levels up to 180 mg/kg can play a major role as an alternative to dietary antibiotics, in improving the productive performance, semen quality, testosterone levels, and the kidney and liver functions in California male rabbits.

## 1. Introduction

Recently, the rabbit industry has played a major role in meeting the increasing requirements of animal protein for human consumption and is becoming an important source of the national economies in Egypt [1,2]. Rabbit meat is characterized by a high protein and low fat and cholesterol content [1]. These nutritional qualities are of great value to the meat industry and consumers. A ban on antibiotic growth promoters by several countries and the menace of antibiotic-resistant bacteria have forced researchers to look for alternatives for improving efficiency in animal production [2,3]. Due to this ban, much research has been conducted in order to explore the use of phytogenic substances as alternate feed additives in animal nutrition [4,5]. Phytogenic substances are generally considered safe and are frequently utilized in the food and feed industries [6].

The effects the phytogenic substances have on the intestinal, antioxidant status, and antimicrobial activity are considered essential for the biological activities. Moreover, the thyme essential oil (TEO), as a phytogenic feed additive, can influence the rabbit performance and welfare positively [7]. Thyme (*Thymus vulgaris*) is an aromatic plant that belongs to the Lamiaceae family, and great attention has been paid to its pharmaceutical and therapeutic effects. Furthermore, supplementation of dietary thyme extract at 0.5 g/kg significantly improved gut integrity and antioxidant status of rabbits [8] mainly because of the thyme active components. However, other studies have not observed any effect of the dietary inclusion of thyme on growth development [9] or the carcass of young rabbits [10]. The main components of TEO are thymol, carvacrol, γ-terpinene, p-cymene, β-myrcene, linalool, and terpinen-4-ol [1,11]. These active components had the best oxidative status [12] and may reduce serum cholesterol [13], which could improve the reproductive performance of rabbits. El-Ratel et al. [14] reported that oral administration of phytobiotics improved the liver function of rabbits compared to the control. Similarly, supplementation of 2.5% of thyme leaves to rabbit diets remarkably enhanced kidney function by decreasing urea and creatinine levels [15]. However, no information is available regarding the effects of TEO, as alternatives to dietary antibiotic growth promoters, on the performance, semen quality, kidney and liver functions, and testosterone levels in male rabbits. To explore the effects of TEO on the productive and physiological status of male rabbits, we investigated the efficiency of TEO as an alternative to antibiotics for improving the productive performance, testosterone levels, and liver and kidney functions of male rabbits.

## 2. Materials and Methods

### 2.1. Animals and Housing

A total of one hundred and fifty, 70-day-old male Californian rabbits, weighing 1250 ± 30 g, were utilized in this study. The rabbits were acquired from a Breeding Agricultural Research Centre, Faculty of Agriculture, South Valley University, Egypt. The Institutional Animal Care and Use Committee of University of South Valley University approved the experimental protocol used in this study in accordance with the guidelines of the Egyptian Research Ethics Committee and the guidelines in the Guide for the Care and Use of Laboratory Animals (2011). All procedures in the current study were in accordance with the European Union Directive 2010/63/EU regarding the protection of animals utilized in experimentation. Rabbits were housed separately in cages (one animal per cage) of galvanized wire net (width × length × height: 60 cm × 60 cm × 40 cm), equipped with an automatic drinker and a manual feeder. Farm temperature was maintained at 23 °C, and the rabbits were exposed to a cycle of 16 h of light and 8 h of darkness during the experimental period. Fresh tap water was available ad libitum via stainless steel nipples located inside each cage. The study was performed following the ARRIVE guidelines for the reporting of animal experiments [16]. Rabbits were housed under the same managerial, hygienic, and housing conditions during the whole experimental period. The health condition of all the rabbits was closely monitored through daily health checks. After the experimentation, all the remaining animals were released.

### 2.2. Experimental Diets and Growth Performance

The male Californian rabbits were assigned to five dietary treatment groups of 30 rabbits each. Dietary treatments included a basal diet as a negative control (NC), a basal diet supplemented with a 500 mg/kg oxytetracycline antibiotic as a positive control (PC), and a basal diet supplemented with 60, 120, or 180 mg/kg of TEO. The experiment period lasted for 60 days. Table 1 presents the experimental ration ingredients and chemical composition. The diets were formulated to contain adequate levels of nutrients for rabbits as per the National Research Council (NRC) [17]. Rabbits were individually identified, and their body weight (BW) values were recorded at day 0 (70 days old), mid-experiment (100 days old), and end of the experiment (130 days old). Daily body weight gain (DBWG) was calculated for each period per pen. Additionally, the feed consumption for each pen between weighing was determined through the measurement of feed residue on the same day as the rabbits were weighed. Feed conversion ratio (FCR) was calculated as feed per gain based on the weight of feed consumed divided by DBWG per pen. Besides, mortality was observed daily during the entire experimental period.

### 2.3. Chemical Analysis

The feed was analyzed for moisture by oven drying (method no. 930.15), ash by incineration (method no. 942.05), protein by Kjeldahl (method no. 984.13), ether extract by Soxhlet fat analysis (method no. 920.39), and calcium and phosphorus (Ca and P; method no. 999.10) as described by the AOAC International [18]. Lysine and, after performic acid oxidation in 6 M HCl for 24 h at 100 °C under an N atmosphere, and methionine were determined as methionine sulfone after cold performic acid oxidation overnight and hydrolyzing with 7.5 N HCl at 110 °C (procedure 4.1.11; alternative 1; AOAC, 2000) [18] for 24 h, followed by analysis using an amino acid analyzer (Hitachi L-8800, Tokyo, Japan).

The gross energy (GE) contents of the diet and feces were measured using an adiabatic bomb calorimeter (Parr Instrument Company, Moline, IL, USA). A digestibility trial was conducted using thirty 100-day-old male Californian rabbits to determine the dry matter (DM) and digestible energy (DE) of the basal diet according to Perez et al. [19]. Rabbits were housed in individual metabolism cages (measuring width × length × height to be 50 cm × 60 cm × 40 cm). A 10-day adaptation period was followed by continuous feces collection for 5 consecutive days (collection period). Samples of daily feces of each rabbit were taken and oven dried at 60 °C for 48 h, then was ground and stored for proximate chemical analysis. Chemical analyses for the digestibility trial (DM and GE of diets and feces) were conducted at the Animal Nutrition Laboratory, Faculty of Agriculture, South Valley University, Egypt. The DE refers to GE intake minus energy lost in feces according to Hall et al. [20].

Analysis of the chemical composition of hydrodistilled TEO (Table 2) was conducted using a gas chromatography–mass spectrometry (GC/MS) system as per Abozid and Asker [21]. The TEO was purchased from El Hawag Natural Oils Company, Cairo, Egypt. The TEO was extracted by hydrodistillation in a Clevenger-type apparatus for three hours. The TEO was analyzed at the Department of Medicinal and Aromatic Plants Research, National Research Centre, Egypt, by gas chromatography (Delsi 121C gas chromatograph). Constituents were identified by GC/MS, using a Sigma 300 apparatus attached to an HP 5970 300 mass spectrometer.

### 2.4. Semen Collection and Assay

Fifteen male rabbits from each group were selected for semen characterization, including volume of each ejaculate, sperm livability, sperm motility, abnormal sperm, and sperm forward motility of sexually mature rabbits (130 days of age), which were assessed as described previously by Abdel-Wareth et al. [4] and El-Desoky et al. [22].

Assessments of live, dead, and abnormal sperms were performed by counting 200 sperm cells using an Eosin–Nigrosin staining mixture. Complete or partial, purple-stained sperm cells were considered nonviable, whereas nonstained sperm cells were considered viable. Percentages of motile sperms on a warm stage showing progressive forward movement were visually calculated in several microscopic fields under 100× magnification using light microscope.

### 2.5. Blood Biochemical Assay

At the end of the experimental period, rabbits were fasted 12 h prior to blood sampling. The treated animals were anesthetized by intramuscular injection of ketamine and xylazine, and then 5 mL of blood was withdrawn from one of the external ear veins. Blood samples were centrifuged at 3000 rpm for 15 min, where the serum was collected and stored at −20 °C until assayed for biochemical analysis. Serum testosterone concentrations were measured by immunoassay using commercial kits (Monobind, Inc., Lake Forest, CA 92630, USA). Liver enzymes, alanine transaminase (ALT) and aspartate transaminase (AST); the kidney function markers, creatinine and urea, concentrations, were also measured using standard diagnostic kits (Monobind, Inc., Lake Forest, CA 92630, USA).

### 2.6. Statistical Analysis

The statistical analysis was conducted by ANOVA followed by Duncan’s test using SAS software [23]. The cage was the experimental unit for each parameter. The significance effects were declared at *P* < 0.05. Orthogonal polynomial contrasts were also utilized for determining the linear and quadratic effects of levels of TEO inclusion considering only negative control (0 mg/kg TEO) as a control, whereas positive control was not included in this analysis. Significance was declared at *P* ≤ 0.05.

## 3. Results

### 3.1. Growth Performance

Table 3 presents the effects of TEO levels, as an alternative to dietary antibiotics, on growth performance. The BW significantly increased (*P* < 0.001) with the increasing levels of TEO supplementations at 100 and 130 days of age. There was no significant difference on initial BW at 70 days of age among treatments. Furthermore, the TEO supplementations at 60, 120, 180 mg/kg increased DBWG and improved FCR of male Californian rabbits compared to the PC and NC groups during the periods of 70–100, 100–130, and 70–130 days of age. The highest DBWG and most efficient FCR were found in the 180 mg, 120 mg, and 60 mg TEO groups, then the PC and NC. The weakest performance was recorded for the NC group compared with PC and TEO groups. In terms of the daily feed intake (DFI), the highest DFI was observed (*P* < 0.001) in 180 and 120 mg TEO supplementations compared with 60 mg TEO, NC, and PC groups during the periods of 70–100 and 70–130 days of age.

On the other hand, the supplemented TEO groups did not affect DFI (*P* ≥ 0.05) compared to control groups during the period of 70–100 days of age.

### 3.2. Semen Characteristics

Table 4 displays the effects of TEO on semen characteristics. Results demonstrated that TEO increased the sperm livability, sperm motility, and ejaculate volume compared with PC and NC groups at the end of treatments. Abnormal sperm was reduced (*P* < 0.01) as TEO increased, compared to PC and NC groups. Moreover, supplementation of TEO up to 180 mg/kg to male rabbit diets remarkably increased (*P* < 0.001) sperm forward motility% and sperm livability% in comparison with control groups. However, no significant difference was observed in the semen pH values between treatments. Overall, the PC (oxytetracycline) exhibited a significant increase in semen quality compared to NC.

### 3.3. Blood Biochemical Constituents

**Figure 1:**
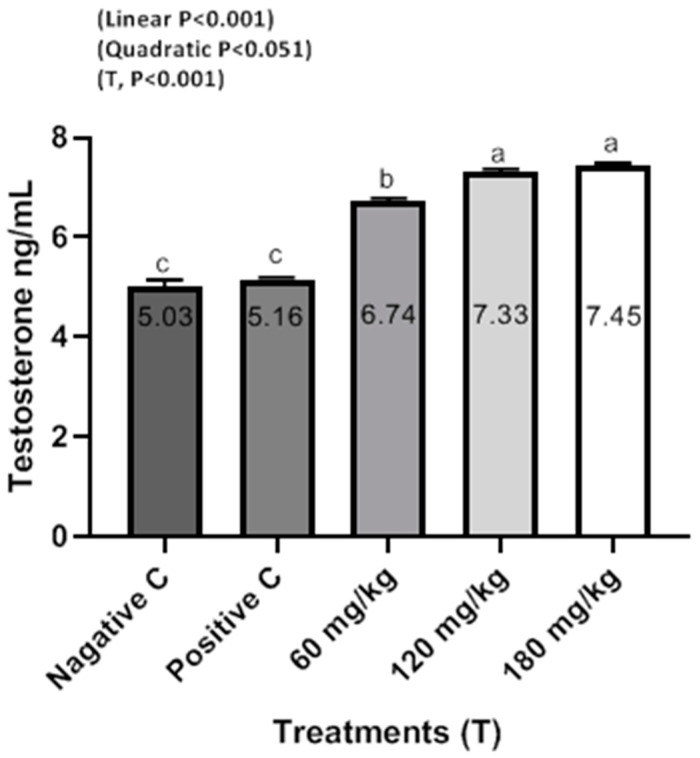
Testosterone ng/mL of male rabbits in response to dietary treatments including a basal diet (NC), a basal diet supplemented with a 500 mg/kg oxytetracycline antibiotic (PC), and a basal diet supplemented with 60, 120, or 180 mg/kg of TEO at 130 days of age. ^a–c^ Means within a row with different superscripts differ significantly at *P* < 0.05.

**Figure 2:**
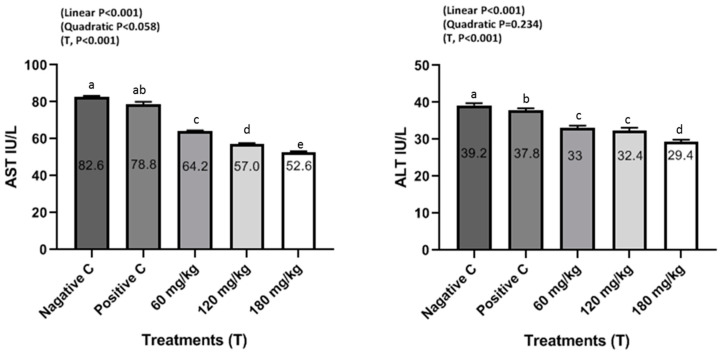
Liver enzyme (alanine transaminase (ALT) and aspartate transaminase (AST)) levels of male rabbits in response to dietary treatments including a basal diet (NC), a basal diet supplemented with a 500 mg/kg oxytetracycline antibiotic (PC), and a basal diet supplemented with 60, 120, or 180 mg/kg of TEO at 130 days of age. ^a–e^ Means within a row with different superscripts differ significantly at *P* < 0.05.

**Figure 3:**
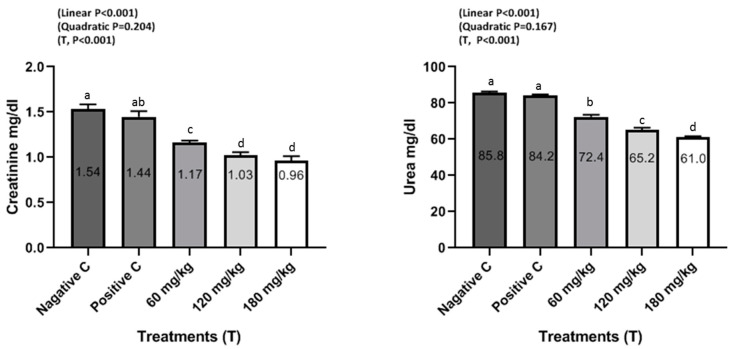
Serum creatinine mg/dl and urea mg/dl of male rabbits in response to dietary treatments including a basal diet (NC), a basal diet supplemented with a 500 mg/kg oxytetracycline antibiotic (PC), and a basal diet supplemented with 60, 120, or 180 mg/kg of TEO at 130 days of age. ^a–d^ Means within a row with different superscripts differ significantly at *P* < 0.05.

The effects of dietary supplemental TEO on blood serum constituents of male rabbits are shown in Figure 1, Figure 2 and Figure 3. Rabbits fed the diets supplemented with TEO at 60, 120, and 180 mg/kg had significantly (*P* < 0.001) reduced serum urea and creatinine compared to PC and NC groups. Moreover, supplementation of TEO to diets of male rabbits significantly (*P* < 0.05) reduced the activity of serum ALT and AST in comparison with PC and NC groups. Furthermore, male rabbits fed diets supplemented with the TEO up to 180 mg/kg exhibited the highest improvement in serum testosterone concentrations (*P* < 0.05) compared to PC and NC groups.

## 4. Discussion

It is extremely difficult to compare the studies that applied different essential oils because the results will be based on numerous factors, including essential composition, doses, extract methods and application, animal age, and housing conditions. The TEO feed additive, as an alternative to dietary antibiotics, is highly safe and could be utilized for improving the productive and reproductive performance of rabbits. The effects the phytogenic substances have on the intestinal health, antioxidant status, and antimicrobial activity are considered essential for the biological activities; however, studies lack the determination of active components, and their mechanisms are still not clear [24,25,26]. Therefore, more studies under standardization are required to explore the mechanisms of TEO on rabbit production and reproduction. In the present study, the main active component of TEO was thymol which constitutes 40% of its analyzed composition. The composition of TEO used in the present study was consistent with that reported in the literature [1,11]. TEO levels caused the highest DFI and the lowest FCR which could be associated with the ability of TEO to improve nutrient absorption. In the current study, it is observed that dietary TEO enhanced the productive performance associated with the present active component, thymol. The current study demonstrated that DBWG and FCR were improved in rabbits fed a control diet supplemented with TEO up to 180 mg/kg compared to PC and NC groups. These improvements may be because the active compounds (thymol and carvacrol) stimulated digestive enzymes, thus leading to the improvement in nutrient digestibility [5,27]. Thymol stimulates the appetite, and the secretion of endogenous digestive enzymes and nutrient absorption mainly because thymol could protect the microvilli [28]. Supplementation of TEO with olive oil enhanced the growth performance of male Californian rabbits under high-temperature environments [1]. Similarly, oral administration of aqueous thyme extract with 50 mg/kg BW improved (*P* < 0.001) BWG, FI, and FCR of rabbits compared to the control [29].

The TEO levels significantly increased the reproductive performance of male rabbits at the end of the treatments period (Table 4). Interestingly, the improvements in the reproductive performance of male rabbits were in parallel with those of testosterone concentration (Figure 1). Ruiz-Olvera et al. [30] demonstrated that semen volume and sperm motility are associated with serum testosterone concentrations. Supplementation of thyme aqueous extracts significantly increased the semen quality of rabbits [31]. In addition, semen volume, sperm motility, sperm concentration, and sperm livability significantly increased (*P* < 0.05) in rabbits received aqueous thyme extract with 50 mg/kg compared to the control [29].

Blood biochemical analysis reflects internal organ integrity. In this direction, this study indicated that TEO did not cause obvious damage to any of the internal organs, including the kidney, liver, and heart. These obtained results can be confirmed by liver and kidney function. In the current study, supplementation of TEO to male rabbits significantly reduced serum urea, creatinine, and ALT and AST activities and increased serum testosterone levels compared to PC and NC groups, but the values were within the normal physiological range [14,16,29,30,31]. These results agree with those of El-Ratel et al. [14], where it is reported that the activity of AST and ALT of rabbits was remarkably enhanced by oral administration of phytogenic additives (5 or 10 mg allicin/BW) compared to the control. Supplementation of 2.5% of thyme leaves to rabbit diets significantly decreased urea and creatinine [15]. Similarly, Abdel-Gabbar et al. [31] demonstrated that thyme extracts at 100 mg/kg induced significant (*P* < 0.001) reduction in creatinine and urea as well as ALT and AST of rabbits compared to the control group. However, Dieumou et al. [32] reported that the administration of garlic and ginger oils via stomach tube in four doses, 0 (control), 10 mg/kg/day, 20 mg/kg/day, and 40 mg/kg/day, showed no clear variations recorded in ALT, AST and serum creatinine features due to ginger treatments. Shanoon et al. [33] indicated that there were no obvious differences in serum ALT, AST and blood creatinine features, indicating that none of the examined three dosages of the oil administered to birds was harmful. In addition, Herve et al. [34] found that the serum features of AST or ALT clearly decreased with 100 or 150 mg/kg BW of ginger roots oil when compared to control data, without any harmful outcomes on feed uptake and BW profits.

Therefore, the effect of TEO on the productive and reproductive performance of rabbits is better known. It increased both productive and reproductive performance and the liver and kidney functions, as observed in the current study.

## 5. Conclusions

Considering the above findings, the levels of TEO up to 180 mg/kg can play a major role in enhancing productive performance, semen quality, testosterone levels, and kidney and liver functions in California male rabbits.

## Figures and Tables

**Table 1:** Ingredients and chemical compositions (as-fed basis) of the control diet fed to rabbits throughout the experiment period.

Ingredients	%	Chemical Analysis	%
Yellow maize grain	32.00	Dry matter	91.40
Wheat bran	20.00	Ash	9.80
Soybean meal (44% CP)	18.00	Crude protein	17.00
Wheat straw	12.00	Crude fiber	12.60
Lucerne hay	5.00	Fat-ether extract	2.90
Rice bran	5.00	Digestible energy (MJ/kg)	9.42
Linseed straw	2.80	Calcium	1.30
Sunflower meal	2.50	Phosphorus	0.86
Limestone	2.00	Lysine	0.60
Sodium chloride	0.30	Methionine	0.41
Vitamin–mineral premix ^1^	0.30		
dl-Methionine	0.10		

^1^ Per kg of ration: vitamin A, 10.000 IU; vitamin D_3_, 900 IU; vitamin E, 50.0 mg; vitamin K, 2.0 mg; vitamin B_1_, 2.0 mg; folic acid, 5.0 mg; pantothenic acid, 20.0 mg; vitamin B_6_, 2.0 mg; choline, 1200 mg; vitamin B_12_, 0.01 mg; niacin, 50 mg; biotin, 0.2 mg; Cu, 0.1 mg; Fe, 75.0 mg; Mn, 8.5 mg; Zn, 70 mg.

**Table 2:** Major chemical compounds of hydrodistilled thyme essential oil (TEO) as detected by gas chromatography–mass spectrophotometer (GC-MS).

Chemical Compounds	Rt.	%	Mol. Weight (gm/mol)	Chemical Formula
p-Cymene	6.99	23.59	134.218	C_10_H_14_
Β-linalool	9.61	0.74	154.25	C_10_H_18_
Carvone (carvacrol)	15.70	9.80	150.22	C_10_H_14_
Anethole	17.49	2.50	148.2	C_10_H_12_
Thymol	17.70	39.45	150.22	C_10_H_14_
Carvacrol	18.09	2.07	150.217	C_10_H_14_
*trans*-Caryophyllene	22.46	0.98	204.36	C_15_H_24_
γ-Terpinene	25.14	12.49	136.23	C_10_H_16_
Aromadenrene	34.84	2.12	204.35	C_15_H_24_
Ledol	48.66	2.24	222.358	C_15_H_26_

**Table 3:** Effects of thyme essential oil on the productive performance of male rabbits.

Items	Treatments (T)					SEM ^3^	*P* value		
	NC ^1^	PC ^2^	60 mg	120 mg	180 mg		T ^4^	Lin ^5^	Quad ^6^
Body weight, g									
70 d	1255	1259	1258	1249	1253	6	0.614	0.882	0.263
100 d	1700 ^d^	1751 ^c^	1807 ^b^	1819 ^a^	1835 ^a^	14	<0.001	0.003	0.303
130 d	2288 ^d^	2354 ^c^	2428 ^b^	2441 ^a^	2469 ^a^	21	<0.001	<0.001	0.053
Daily body weight gain, g									
70–100 d	14.85 ^d^	16.40 ^c^	18.33 ^b^	19.02 ^ab^	19.41 ^a^	0.22	<0.001	<0.001	< 0.001
100–130 d	19.59	20.10	20.67	20.71	21.14	0.38	0.076	0.006	0.640
70–130 d	17.22 ^b^	18.25 ^b^	19.50 ^a^	19.87 ^a^	20.28 ^a^	0.25	<0.001	<0.001	0.033
Daily feed intake, g									
70–100 d	54.46 ^c^	55.43 ^c^	57.64 ^b^	58.61 ^ab^	59.65 ^a^	0.51	<0.001	<0.001	0.574
100–130 d	65.20	62.77	63.04	64.48	64.71	0.64	0.053	0.719	0.013
70–130 d	59.83 ^c^	59.10 ^c^	60.34 ^bc^	61.54 ^ab^	62.18 ^a^	0.43	<0.001	<0.001	0.110
Feed conversion ratio									
70–100 d	3.67 ^a^	3.38 ^b^	3.15 ^c^	3.08 ^c^	3.07 ^c^	0.048	0.001	<0.001	<0.001
100–130 d	3.33 ^a^	3.13 ^b^	3.05 ^b^	3.12 ^b^	3.06 ^b^	0.056	0.014	0.006	0.046
70–130 d	3.48 ^a^	3.24 ^b^	3.09 ^c^	3.09 ^c^	3.07 ^c^	0.042	0.001	<0.001	0.002

^a–d^ Means within a row with different superscripts differ significantly at *P* < 0.05. ^1^ NC: basal diet as a negative control. ^2^ PC: a basal diet was supplemented with an antibiotic as a positive control. ^3^ SEM: standard error of means. ^4^ T: NC, PC, and thyme essential oil treatments. ^5,6^ Lin and Quad: linear and quadratic responses, respectively, to thyme essential oil inclusion level considering NC (0 mg/kg thyme essential oil) as a control; PC was not included in this analysis.

**Table 4:** Effects of thyme essential oil on semen quality of male rabbits at 130 days of age.

Items	Treatments (T)					SEM ^1^	*P* Value		
	NC ^1^	PC ^2^	60 mg	120 mg	180 mg		T ^4^	Lin ^5^	Quad ^6^
Semen quality									
Semen volume, ml	0.52 ^d^	0.56 ^c^	0.61 ^b^	0.64 ^a^	0.65 ^a^	0.09	0.001	0.001	0.004
Abnormal sperm, %	16.7 ^a^	15.4 ^ab^	12.6 ^bc^	11.9 ^cd^	11.2 ^d^	0.41	0.001	0.001	0.432
Live sperm, %	75.0 ^d^	75.4 ^d^	79.2 ^c^	81.6 ^b^	83.6 ^a^	0.63	0.001	0.001	0.458
Semen pH, value	7.11	7.09	7.10	7.11	7.10	0.03	0.990	0.965	0.853
Forward motility, %	55.1 ^c^	55.3 ^c^	65.7 ^b^	69.5 ^a^	70.8 ^a^	0.57	0.001	0.001	0.056

^a–d^ Means within a row with different superscripts differ significantly at *P* < 0.05. ^1^ NC: basal diet as a negative control. ^2^ PC: a basal diet supplemented with an antibiotic as a positive control. ^3^ SEM: standard error of means. ^4^ T: NC, PC, and thyme essential oil treatments. ^5,6^ Lin and Quad: linear and quadratic responses, respectively, to thyme essential oil inclusion level considering NC (0 mg/kg thyme essential oil) as a control; PC was not included in this analysis.

## Abbreviations

TEO: thyme essential oil; NC: negative control; PC: positive control; BW: body weight; BWG: body weight gain; FCR: feed conversion ratio; AST: aspartate transaminase; ALT: alanine transaminase.
